# Association between Following the ESCMID Guidelines for the Management of Candidemia and Mortality: A Retrospective Cohort Study

**DOI:** 10.3390/jof8050541

**Published:** 2022-05-23

**Authors:** Charles Maurille, Julie Bonhomme, Anaïs R. Briant, Jean-Jacques Parienti, Renaud Verdon, Anna Lucie Fournier

**Affiliations:** 1 Department of Infectious Diseases, CHU de Caen Normandie, UNICAEN, Normandie University, 14000 Caen, France; verdon-r@chu-caen.fr (R.V.); fournier-an@chu-caen.fr (A.L.F.); 2 INSERM U1311 DynaMicURe, UNICAEN, UNIROUEN, Normandie University, 14000 Caen, France; parienti-jj@chu-caen.fr; 3 Department of Microbiology, CHU de Caen Normandie, UNICAEN, Normandie University, 14000 Caen, France; bonhomme-julie@chu-caen.fr; 4 ToxEMAC-ABTE, UNICAEN, UNIROUEN, Normandie University, 14000 Caen, France; 5 Department of Biostatistics and Clinical Research, CHU de Caen Normandie, 14000 Caen, France; briant-a@chu-caen.fr; 6 Department of Biostatistics and Clinical Research, CHU de Caen Normandie, UNICAEN, Normandie University, 14000 Caen, France

**Keywords:** candidemia, *Candida* spp., fungemia, guidelines

## Abstract

Objectives: The objective of this study was to evaluate the association between ESCMID adherence and 30-day mortality in candidemia. Methods: We performed a retrospective cohort study in two French tertiary-care hospitals. All patients with at least one positive blood culture (BC) for *Candida* spp. between January 2013 and December 2019 were included. An adherent case was defined as a candidemia case for which the treatment fulfilled a bundle of defined criteria based on the latest ESCMID recommendations. We explored factors associated with adherence to ESCMID recommendations in an unadjusted model, and we used a propensity score method to address potential channeling biases with regard to 30-day mortality. Results: During the study period, 165 cases of candidemia were included. Among the ESCMID criteria, funduscopic examination was not performed in 45% and neither was echocardiography in 31%, while the ESCMID criteria were fully implemented in 44 cases (27%). In the propensity score analysis, the all-cause 30-day mortality rate was significantly lower among adherent cases (3.4/36.6, 9%) than among nonadherent cases (42.4/119.5, 36%) (OR = 5.3 95% CI [1.6–17.1]). Conclusions: In our study, adherence to the bundle of criteria for candidemia management was associated with increased survival, supporting additional efforts to implement these recommendations.

## 1. Introduction

Candidemia is a bloodstream infection (BSI) caused by the *Candida* species, and it accounts for 3 to 13% of nosocomial BSIs [1,2]. A trend of increasing cases of candidemia has been recorded since the 2000s in European hospitals [3]. Although the prognosis of patients with candidemia is largely related to host factors, the crude mortality rate is high and ranges from 40% to 55% [4,5]. Moreover, candidemia imposes a health burden with significant increases in the length of stay (LOS) and the cost of hospitalization [6].

The European Society of Clinical Microbiology and Infectious Diseases (ESCMID) published recommendations on the management of candidemia in non-neutropenic and neutropenic patients in 2012 [7,8]. Due to the increasing level of resistance to fluconazole and evidence that echinocandins are more effective than fluconazole, the ESCMID recommended the use of echinocandin in all patients with candidemia; the removal of indwelling lines as soon as possible; the performance of a complementary assessment with transesophageal echocardiography (TOE) and fundoscopy to search for organ involvement; the administration of treatment for a duration of 14 days after the end of candidemia unless organ involvement is found; and the performance of a systematic sequence of blood cultures (BC) to ensure fungal clearance. The Infectious Diseases Society of America (IDSA) also issued recommendations on the management of candidemia in 2016, which contained proposals similar to those issued by the ESCMID [9].

Compliance with recommendations is often weak in audit studies [10]. A few studies focused on adherence to the ESCMID candidemia management recommendations and found conflicting results, especially regarding their impact on mortality [11,12,13,14,15]. The objectives of this study were to audit the management of candidemia, according to the ESCMID recommendations and to evaluate the association between ESCMID adherence and 30-day mortality in two French tertiary-care hospitals.

## 2. Materials and Methods

### 2.1. Study Design and Participants

We conducted a retrospective cohort study in the University Hospital of Caen and the Comprehensive Cancer Centre François Baclesse of Caen. All patients with at least one positive BC for *Candida* spp. between 1 January 2013 and 31 December 2019 were included. Patients under 18 years of age, patients in palliative care, and patients who died within 24 h after the diagnosis of candidemia were excluded. We also excluded patients with an isolated BC containing *C. parapsilosis*, which is commensal of the skin flora of human and it is difficult to differentiate a contaminant from a true pathogen. Only the first episode of candidemia was considered for each patient. Each patient was followed until May 2020. The ethics committee of the biomedical research institute of Caen, Normandy approved the study (ID605, 9 December 2019).

### 2.2. Data Collection

We retrospectively collected demographic data (age, sex, year of candidemia diagnosis), acute and chronic comorbidities (diabetes mellitus, chronic kidney disease, liver cirrhosis, chronic obstructive pulmonary disease (COPD), connective tissue disease, solid malignancy, hematological malignancy, solid organ transplant (SOT), hematopoietic stem cell transplantation (HSCT), human immunodeficiency virus (HIV) infection), clinical characteristics and underlying conditions (department; primary source; presence of a central line, central venous catheter (CVC) or PICC-line/Midline; neutropenia, which was defined as <500 neutrophils per mm^3^; recent antifungal or antibiotic exposure; undernutrition; recent surgery (<1 month); illicit intravenous drug use; concomitant BSI; LOS before candidemia; vein thrombosis; endocarditis; ocular candidiasis), microbiological data (species, resistance to fluconazole or echinocandins), candidemia management (early treatment, type of antifungal, loading dose, duration of treatment, removal of catheter, daily BC, echocardiography, ophthalmologic exam, de-escalation, infectious disease consultation [IDC]), and outcome (mortality, LOS, time to resolution of candidemia).

Electronic medical records (Usv2-Crossway, McKesson, Irving, TX, USA) and the laboratory information system (TDNexLabs, Technidata, France) were prospectively completed by medical and paramedical staff. Anonymized data were used for the study.

### 2.3. Outcome and Definitions

A case considered adherent to the ESCMID recommendations was defined as one in which the candidemia was managed with all the following criteria fulfilled: the administration of an antifungal therapy using an echinocandin or liposomal amphotericin B within 24 h after the diagnosis; the use of a loading dose, if applicable; a duration of treatment lasting at least 14 days following a negative BC; the removal of any central venous catheter (CVC) within 72 h after the diagnosis; the performance of echocardiography and a funduscopic examination within 7 days; and the performance of daily BC monitoring until negative results were obtained [7]. Because it is not always possible to perform TOE, transthoracic echocardiography (TTE) was an acceptable alternative. The administration of liposomal amphotericin B was considered an appropriate treatment in case of neutropenia, suspected endocarditis, or endophthalmitis. Despite kidney toxicity, the efficiency of liposomal amphotericin B and echinocandins are similar, and thus this treatment is recommended as a second-line option in the ESCMID recommendations (grade BI). An antifungal stewardship program with an IDC was implemented in our hospital between April 2014 and December 2015. Outside this period, the request for an IDC was made at the discretion of the physician in charge of the patient.

The time to administer antifungal therapy was determined as the time between the first positive BC and the administration of the first dose of an antifungal drug. The primary source was determined by considering the clinical, radiological and biological records after an analysis of the case performed by an infectious disease specialist. Recurrent candidemia was defined by a positive BC with the same pathogen within 100 days after the first episode and after the end of antifungal treatment. Persistent candidemia was characterized by positive BCs over the course of at least 10 days between the first and the last positive BC. Crude mortality was evaluated 8, 30, and 90 days after the diagnosis of candidemia.

### 2.4. Microbiological Methods

The microbiology laboratory received all BCs from both centers. The blood samples were processed using the BacT/Alert^®®^ system (bioMérieux, Marcy L’Etoile, France) until 2017 and the BacT/Alert^®®^ Virtuo^®®^ system (bioMérieux, Marcy L’Etoile, France) after 2017, with an incubation period of 5 to 8 days. In the case of a positive BC, direct microscopic examination was performed. Subcultures were performed on Sabouraud agar medium (Oxoid) and/or chromogenic medium (CAN2 ChromID Candida, bioMérieux, Marcy L’Etoile, France). Species identification was carried out using a MALDI-TOF mass spectrometer (Microflex LH/SH analyzer, Brucker, Billerica, MA, USA). The minimum inhibitory concentrations (MICs) of the isolates were assessed by the gradient diffusion method with Etests (bioMérieux, Marcy L’Etoile, France), as recommended. The MICs were evaluated according to the European Committee on Antimicrobial Susceptibility Testing (EUCAST) [16].

### 2.5. Statistical Analysis

We estimated the prevalence rate of candidemia as the number of patients who had at least one diagnosis of candidemia divided by the number of patients admitted during the study period. Continuous variables are reported as medians with interquartile ranges (IQRs) or means and standard deviations (SDs), as appropriate. Dichotomous and categorical variables are presented as the number and percentage of the study population.

**Figure A2:**
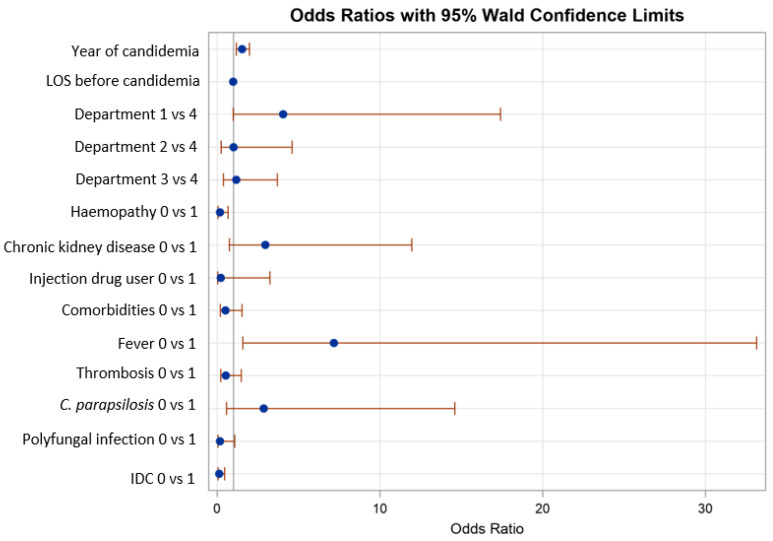
Multivariate analysis of factors associated with ESCMID nonadherence. Abbreviations: Department 1, medical ICU; Department 2, surgical ICU; Department 3, medical; Department 4, surgical; IDC, infectious disease consultation; LOS, length of stay.

We explored the factors associated with adherence to the ESCMID recommendations in an unadjusted model using chi^2^ or Fisher’s exact tests for qualitative variables and Student’s *t* tests or Mann–Whitney U tests for quantitative variables. Baseline differences between groups that were or were not adherent to the ESCMID recommendation may have been present because the two groups were not randomized. Therefore, we analyzed our data using a propensity score method. The inverse probability weighting treatment (IPWT) strategy was applied to address the potential channeling bias with regard to the outcomes. We modelled the probability of ESCMID adherence using a nonparsimonious logistic regression with the following variables: age, year, department, LOS before candidemia, abdominal surgery, solid malignancy, abdominal tumor, hematological malignancy, neutropenia, chronic kidney disease, COPD, illicit intravenous drug, at least one comorbidity, CVC, fever, vein thrombosis, cutaneous primary source, *C. parapsilosis*, other species, 4olyfungal infection, and IDC (see Figure A2). We used IPWT to weight the chi^2^ or Fisher’s exact test and Student’s *t* test or the Mann–Whitney U test (stabilized weights) for 30-day mortality and LOS (respectively).

After adjusting with the IPWT method, we compared the baseline characteristics using chi^2^ or Fisher’s exact tests for categorical variables and Student’s *t* test or Mann–Whitney U tests for continuous variables according to their distribution with stabilized weight.

A *p* value less than 0.05 was considered significant; all *p* values were two-tailed. No adjustment for multiple comparisons was performed. Statistical analyses were performed using Stata 14.0 software (Stata Corporation, College Station, TX, USA); SAS statistical software, version 9.4 (SAS Institute Inc., Cary, NC, USA); and R software, version 3.6.2 (The R Foundation for Statistical Computing, Vienna, Austria).

## 3. Results

During the study period, we identified 209 candidemia cases that occurred in 207 patients, corresponding to a prevalence of 0.26 per 1000 admissions. After the exclusion of 29 patients who died within 24 h after diagnosis, 7 patients in palliative care, 5 patients under 18 years of age and 1 patient with a unique BC that was positive for *C. parapsilosis*, 165 patients were included in the study. The demographic data, underlying conditions, clinical characteristics, microbiological data, and outcomes for those 165 patients are described in Table 1.

### 3.1. Patient Characteristics

The median age was 66 years (IQR 56–76), and 107 (65%) were males. At least one comorbidity was found in 127 (77%) patients. Seventy-two patients (44%) had a solid malignancy with active treatment within one year. One hundred thirty-nine (84%) had a CVC. At the time of BC taking, 58 (35%) were in an intensive care unit (ICU).

### 3.2. Microbiology

The most frequent species isolated were *C. albicans* (61%), *C. glabrata* (13%), and *C. parapsilosis* (10%). *C. albicans*, *C. glabrata*, and *C. krusei* were resistant to fluconazole in 1% (*n* = 1), 18% (*n* = 4) and 100% (*n* = 5) of cases, respectively. No strain of *C. parapsilosis* or *C. tropicalis* was resistant to fluconazole. Strains not susceptible to echinocandins were found in 0% of *C. albicans*, 5% of *C. glabrata* (*n* = 1), 100% of *C. parapsilosis* (*n* = 18), 10% of *C. tropicalis* (*n* = 1), and 20% of *C. krusei* (*n* = 1) isolates. Eight patients (5%) had mixed infections with different *Candida* species isolated from blood samples.

### 3.3. Management and Outcomes

**Figure 1:**
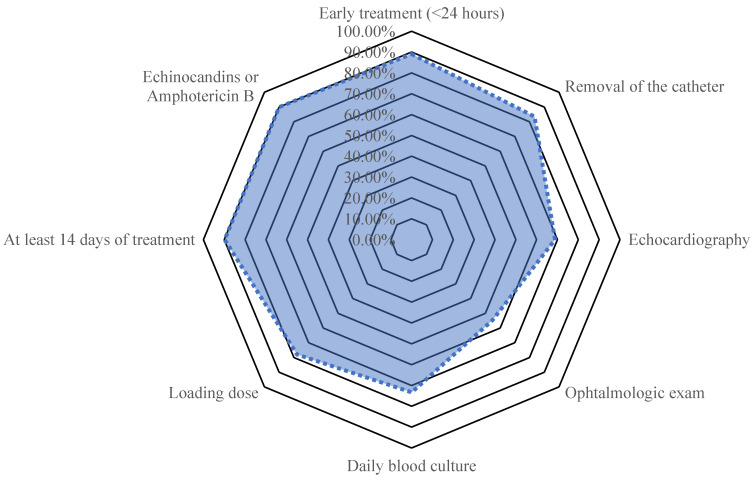
Point-by-point adherence with ESCMID recommendations.

Among the 165 patients included in the cohort, the ESCMID recommendations were fully implemented in 44 (27%). Figure 1 depicts point-by-point adherence to recommendations. Among the ESCMID criteria, funduscopic examination was not performed in 45% (*n* = 71), echocardiography in 31% (*n* = 50), or daily BCs in 27% (44%). The empirical antifungal therapy consisted of echinocandins in 143 (86%), fluconazole in 17 (10%) and liposomal amphotericin B in 5 (3%). Among the 86 patients who underwent dilated fundoscopy, six (7%) were diagnosed with ocular candidiasis. Echocardiography, which was performed for 110 patient, identified 6 (6%) patients with infectious endocarditis.

The mortality rates at 8, 30, and 90 days were 7%, 28%, and 39%, respectively. The median (IQR) LOS was 35 (20–50) days. The median (IQR) time to resolution of candidemia was 2 days [1,2,3].

### 3.4. Adherence to ESCMID Recommendations and Its Association with All-Cause 30-Day Mortality: Propensity Score Analysis

A univariate analysis of factors associated with adherence to the recommendations is presented in Table 2. Factors associated with adherence were the year of onset of candidemia (*p* = 0.015), department (*p* = 0.047), hematological malignancy (*p* = 0.014), and performance of an IDC (*p* = 0.006), while fever was associated with nonadherence (*p* = 0.017). The characteristics of the pseudopopulation based on the propensity score estimated with the IPWT method are presented in Table 3.

Table 4 shows the univariate analysis of 30-day mortality and LOS according to ESCMID adherence in the unadjusted population (Table 4a) and in the pseudopopulation stabilized with the IPWT method (Table 4b). All cause 30-day mortality differed between adherent and nonadherent cases in the unadjusted analysis with 6/44 (14%) deaths among the adherent cases and 40/121 (33%) among the nonadherent cases (OR = 3.1 95% CI [1.2–8.0], *p* = 0.014). After the IPWT method was applied, all-cause 30-day mortality was significantly lower among the adherent cases (3.4/36.6, 9%) than the nonadherent cases (42.4/119.5, 36%) (OR = 5.3 95% CI [1.6–17.1], *p* = 0.004). The median LOS was longer among the adherent cases (38 versus 30 days), but the difference was not significant (*p* = 0.09).

## 4. Discussion

Among the 165 patients with candidemia, adherence to the ESCMID recommendations was low, despite the associated mortality. Importantly, after carrying out a propensity score analysis, we showed a significantly lower rate of mortality when the recommendations were fully applied.

A case was defined as adherent when eight prespecified key items belonging to the ESCMID recommendations were fulfilled. While some items are uniformly recognized by the main recommendations as being essential elements for the management of candidemia (early treatment, removal of the catheter), other items are not standardized (type of primary treatment, step-down therapy, funduscopic exam), leading to uncertainties in clinical decision-making [10].

Recently, the European Confederation of Medical Mycology has designed a score, called the EQUAL Candida Score, that can be used to assess the overall management of candidemia [17]. It weights and it adds the strongest recommendations of the IDSA and the ESCMID, including initial blood culture volume, species identifications, susceptibility testing, echocardiography, fundoscopy, echinocandin treatment, step down to fluconazole depending on susceptibility result, at least 14 day of treatment after first negative blood culture, removal of the CVC, and follow-up blood culture. Since then, several studies have shown a decrease in mortality in patients who were managed with greater adherence to the recommendations according to the EQUAL score [11,12,14]. Another score, developed by the Spanish study group focused on fungal infections called GEMICOMED, analyzing nine selected recommendations from the IDSA and ESCMID: early appropriate antifungal therapy, use of echinocandin or amphotericin B therapy in patients with neutropenia or septic shock, administration of a different class of antifungal drug in patients with breakthrough candidemia, catheter removal, follow-up blood cultures, performing an ophthalmoscopic examination, an echocardiography, and treatment duration according to the complexity of the infection [15]. After using a propensity score analysis, adherence to less than 50% of the nine items proved to be an independent risk factor for mortality, which is consistent with our results. Compared with the EQUAL score, we did not include microbiological data to define good compliance but we added the earliness of the first antifungal infusion and loading dose. For blood culture, volume is not recorded in the computer record so we cannot evaluate this data. Compared to our score, the loading dose was not included in the study conducted by GEMICOMED and the definition of adapted treatment differed.

Only 68.8% (*n* = 110) of our cohort underwent echocardiography, of whom six (5.5%) were diagnosed with endocarditis. Endocarditis is a serious complication of candidemia, and it occurs in 2.5% to 4.2% of all candidemia cases [18,19,20]. The cost-effectiveness of systematic echocardiography is currently debated due to the low rate of endocarditis in candidemia patients, the low sensitivity of TTE and the invasiveness of TOE [20]. Current recommendations for performing echocardiography differ. The recommendations from the IDSA do not discuss echocardiography [9]. In the recommendations from the German-Speaking Mycological Society and the Paul Ehrlich Society for Chemotherapy, echocardiography is recommended for complicated candidemia, such as in the case of persistent positive BCs [21]. The ESCMID recommends the performance of TOE as soon as possible for all patients with candidemia [7]. Nevertheless, it may be difficult to follow this recommendation for patients who have difficulty swallowing, disturbed consciousness, poor compliance with care, or esophageal varices.

Ocular candidiasis is a hematogenous spread of candidemia. There are two forms of ocular candidiasis: chorioretinitis, which is a lesion restricted to the choroid and retina, and endophthalmitis, which a lesion extending into the vitreous body. Ocular candidiasis may require an aggressive treatment with prolonged systemic antifungals, vitrectomy, and intravitreal injection of amphotericin B [7]. In our study, ocular examination with fundoscopy was performed in only 86 patients (54.8%), leading to a diagnosis of 6 cases of ocular candidiasis (7%). Intraocular candidiasis rates are variable, ranging from 1.3% to 26.5% [22,23]. IDSA and ESCMID recommend a fundoscopic examination for all candidemia to screen for ophthalmologic involvement [7,9]. However, the value of systematic fundoscopy remains controversial because of the limited benefit, especially in asymptomatic patients [24,25].

Our findings should not be interpreted without considering several limitations, which are mainly related to the design of the study. First, it was not a randomized study, so there is the potential for the influence of confounding factors. A propensity score constructed with the IPWT method was used to control for such confounders. However, there may have been confounding factors that were not included in the propensity score. Second, this was a bicentric study, and the epidemiological characteristics and local practices cannot be extrapolated to all settings. Finally, the significant correlation that we demonstrated between the outcome and the adherence to the recommendations should not be misinterpreted. It is difficult to say whether treatment that was adherent to the recommendations directly led to better outcomes, or if adherence to the recommendations was a marker of the global quality of care [26].

Despite these limitations, the strengths of this study deserve to be discussed. The power of the study was sufficient to perform a multivariate analysis and a propensity score analysis, which improved the rigor of the evidence. Data were collected from medical records, yielding a large amount of reliable information with limited missing data points. These medical records provide a longitudinal overview of the care of patients with candidemia in real life over a 6-year period.

## 5. Conclusions

Our study showed the extent of the lack of adherence to the ESCMID recommendations regarding the management of candidemia and the protective effect of adherence against 30-day mortality. More efforts to implement these recommendations are warranted to improve the outcomes of this deadly infection.

## Figures and Tables

**Table 1:** Clinical, microbiological characteristics, treatment and outcome of the cohort.

	*n* = 165 (%)
Year of candidemia:	
2013	16 (9.7)
2014	28 (17.0)
2015	30 (18.2)
2016	29 (17.6)
2017	27 (16.4)
2018	15 (9.1)
2019	20 (12.1)
Age in year, median (IQR)	66 (56–76)
Male sex	107 (64.8)
Department:	
Medical ICU	21 (12.7)
Surgical ICU	37 (22.4)
Medical ward	68 (41.2)
Surgical ward	39 (23.6)
Comorbidities:	
Diabates	32 (19.4)
Chronic kidney disease	29 (17.6)
Liver cirrhosis	10 (6.1)
COPD	4 (2.4)
Connective tissue disease	6 (3.6)
Solid malignancy ^a^	72 (43.6)
Abdominal tumor	39 (23.6)
Hematological malignancy	16 (9.7)
Chemotherapy < 1 month	46 (27.9)
SOT	7 (4.2)
HSCT	5 (3.0)
HIV	1 (0.6)
At least 1 comorbidity	127 (77)
Clinical:	
Surgery < 1 month	81 (49.1)
Abdominal surgery < 1 month	55 (33.3)
Undernutrition	102 (61.8)
Illicit intravenous drug use	3 (1.8)
Neutropenia	10 (6.1)
Concomitant BSI ^b^	88 (53.3)
Prior antibiotic exposure (<5 days)	124 (75.1)
Recent antifungal exposure (<1 month)	13 (7.9)
LOS before candidemia, day, median (IQR)	9 (2–16)
Fever	153 (92.7)
Vein thrombosis	44 (26.6)
Endocarditis ^c^	6 (5.5)
Ocular candidiasis ^d^	6 (7.0)
PICC-line or MID-line	45 (27.3)
CVC	94 (57.0)
Primary source:	
Gastrointestinal	69 (41.8)
Catheter-related	74 (44.8)
Urologic tract	19 (11.5)
Skin	3 (1.8)
Candida species: (*n* = 173)	
C. albicans	105 (60.7)
C. glabrata	22 (12.7)
C. parapsilosis	18 (10.4)
C. tropicalis	10 (5.8)
C. krusei	5 (2.9)
Other species ^e^	4 (2.3)
Polyfungal infection (*n* = 173)	8 (4.6)
Fluconazole resistance (*n* = 173)	11 (6.4)
Echinocandins NS (*n* = 173)	20 (11.6)
Persistent candidemia (>10 days)	25 (15.2)
Recurrent candidemia	10 (6.1)
Item of adherence case:	
Early treatment (<24 h)	147 (89.1)
Echinocandins or amphotericin B	148 (90.0)
Loading dose (if applicable) ^f^	67 (77.9)
At least 14 days of treatment ^g^	123 (89.8)
Removal of catheter (if applicable) ^h^	116 (83.5)
Daily blood culture	121 (73.3)
Echocardiography ^i^ (TTE and TOE)	110 (68.8)
Ophtalmologic exam ^j^	86 (54.8)
Clinical management, others:	
Initial treatment by caspofungin	69 (41.8)
Initial treatment by micafungin	74 (44.2)
Initial treatment by liposomal Amphotericin B	5 (3.0)
Initial treatment by fluconazole	17 (10.3)
No treatment	1 (0.6)
De-escalation (if possible)	105 (65.2)
Antifungal duration, day, median (IQR) ^k^	16 (15–17)
At least 5 days of intravenous treatment	132 (93.0)
IDC	120 (72.7)
Outcomes:	
Adherence with all recommendations	44 (26.7)
LOS, day, median (IQR) ^l^	35 (20–50)
Time to resolution of candidemia, day, median (IQR)	2 (1–3)
8-day mortality	12 (7.3)
30-day mortality	46 (27.9)
90-day mortality	64 (38.8)

Abbreviations: BSI, bloodstream infection; COPD, chronic obstruction pulmonary disease; HIV, human immunodeficiency virus; HSCT, hematopoietic stem cell transplantation; ICU, intensive care unit; IDC, infectious disease consultation; IQR, interquartile range; LOS, length of stay; NS: nonsusceptible; SOT solid organ transplant. Data are presented as absolute numbers (%) unless otherwise indicated. ^a^ Solid malignancy with an active treatment within 1 year. ^b^ Concomitant BSI is characterized by a BSI with bacteria within 14 days before or after the candidemia. ^c^ Data available for 110 patients who benefited an echocardiography. ^d^ Data available for 86 patients who benefited an a fundoscopic examination. ^e^ Others: *C. lusitaniae n* = 4; *C. orthopsilosis n* = 3; *C. kefyr n* = 3; *C. inconspicua n* = 1; *C. dubliniensis n* = 1; *C. nivariensis n* = 1. ^f^ Data available for 86 patients who received caspofungin or fluconazole. ^g^ Data unavailable for 28 patients who died within 14 days. ^h^ Data available for 139 patients who had central venous catheter. ^i^ Data unavailable for 5 patients who died within 7 days. ^j^ Data unavailable for 8 patients who died within 7 days. ^k^ Data unavailable for 31 patients who died before the end of antifungals treatment. ^l^ Data unavailable for 6 patients.

**Table 2:** Unadjusted analysis of factors associated with ESCMID adherence.

	Unadjusted		
	Adherent Case *n* = 44	Nonadherent Case *n* = 121	*p* *
Age in years, mean (SD)	63.1 (12.8)	65.2 (14.1)	0.39
Year, median (IQR)	2017 (2015–2018)	2016 (2014–2017)	0.015
LOS before candidemia, day, median (IQR)	7.5 (2.0–16.5)	10.0 (2.0–25.0)	0.36
Male sex	30 (68)	77 (64)	0.59
Department:			
Medical ICU	9 (20)	12 (10)	0.047
Surgical ICU	5 (11)	32 (26)	
Medical ward	22 (50)	46 (38)	
Surgical ward	8 (18)	31 (26)	
Surgery < 1 month	20 (45)	61 (50)	0.58
Abdominal surgery	11 (25)	44 (36)	0.18
Concomitant BSI	23 (52)	65 (54)	0.87
Solid malignancy	17 (39)	55 (45)	0.44
Abdominal tumor	8 (18)	31 (26)	0.32
Hematological malignancy	9 (20)	7 (6)	0.014
HSCT	2 (5)	3 (2)	0.62
Neutropenia	5 (11)	5 (4)	0.14
SOT	1 (2)	6 (5)	0.68
Diabetes	9 (20)	23 (19)	0.84
Chronic kidney disease	4 (9)	25 (21)	0.11
Liver cirrhosis	3 (7)	7 (6)	0.73
COPD	2 (5)	2 (2)	0.29
Connective tissue disease	1 (2)	5 (4)	1.00
HIV infection	0 (0)	1 (1)	1.00
Illicit intravenous drug use	2 (5)	1 (1)	0.18
At least 1 comorbidity	36 (82)	91 (75)	0.38
Undernutrition	27 (61)	75 (62)	0.95
CVC	35 (80)	104 (86)	0.32
Fever	37 (84)	116 (96)	0.017
Vein Thrombosis	12 (27)	23 (19)	0.26
Primary source:			
Gastrointestinal	18 (41)	51 (42)	
Catheter-related	19 (43)	55 (45)	0.48
Urologic tract	5 (11)	14 (12)	
Skin	2 (5)	1 (1)	
*Candida* species:			
*C. albicans*	28 (64)	75 (62)	
*C. glabrata*	6 (14)	16 (13)	
*C. parapsilosis*	3 (7)	15 (12)	0.58
Other species	10 (23)	18 (15)	
Polyfungal infection	4 (9)	4 (3)	0.22
R to fluconazole or NS to echinocandins	9 (20)	20 (17)	0.56
Recent antifungal exposure (<1 month)	3 (7)	10 (8)	1.00
IDC	39 (89)	81 (67)	0.006

Abbreviations: BSI, bloodstream infection; COPD, chronic obstruction pulmonary disease; CVC, central venous catheter; HIV, human immunodeficiency virus; HSCT, hematopoietic stem cell transplantation; ICU, intensive care unit; IQR, interquartile range; LOS, length of stay; NS, nonsusceptible; SD, Standard Deviation; SOT solid organ transplant. Data are presented as absolute numbers (%) unless otherwise indicated. Variables used in the estimation of the propensity score. * chi^2^ or Fisher’s exact test for qualitative variables and Student’s *t* or Mann-Whitney U test for quantitative variables.

**Table 3:** Weighted pseudo-population of the cohort after stabilized IPWT.

	After Stabilized IPWT		
	Adherent Case wn = 36.6	No Adherent Case wn = 119.5	*p* **
Age in years, mean (SD)	62.2 (10.9)	64.1 (14.0)	0.45
Year, median (IQR)	2016 (2014–2018)	2016 (2015–2017)	0.75
LOS before candidemia, day, median (IQR)	10.0 (2.0–19.0)	9.0 (1.0–25.0)	0.83
Male sex	26.3 (72)	75.9 (63)	0.36
Department:			
Medical ICU	5.4 (15)	14.8 (12)	0.76
Surgical ICU	5.1 (14)	26.3 (22)	
Medical ward	16.5 (45)	49.1 (41)	
Surgical ward	9.7 (26)	29.3 (24)	
Surgery < 1 month	15.6 (42)	52.6 (44)	0.16
Abdominal surgery	11.1 (30)	38.3 (32)	0.85
Concomitant BSI	21.6 (59)	64.0 (54)	0.57
Solid malignancy	20.2 (55)	53.3 (45)	0.26
Abdominal tumor	11.7 (32)	28.9 (24)	0.35
Hematological malignancy	3.5 (10)	9.2 (8)	0.51
HSCT	0.9 (2)	5.6 (5)	1.00
Neutropenia	2.5 (7)	5.8 (5)	0.45
SOT	0.3 (1)	6.9 (6)	0.36
Diabetes	7.1 (19)	21.3 (18)	0.83
Chronic kidney disease	5.1 (14)	21.9 (18)	0.54
Liver cirrhosis	1.6 (4)	8.7 (7)	1.00
COPD	1.7 (5)	2.6 (2)	0.34
Connective tissue disease	0.4 (1)	6.1 (5)	0.34
HIV	0.0 (0)	0.8 (1)	1.00
Illicit intravenous drug use	0.9 (2)	2.9 (2)	1.00
At least 1 comorbidity	29.8 (82)	92.9 (78)	0.63
Undernutrition	26.7 (73)	69.5 (58)	0.11
CVC	30.2 (82)	99.3 (83)	0.93
Fever	32.7 (89)	111.0 (93)	0.49
Vein thrombosis	7.1 (19)	23.4 (20)	0.98
Primary source:			
Gastrointestinal	18.1 (49)	52.5 (44)	
Catheter-related	14.5 (40)	51.2 (43)	0.93
Urologic tract	3.2 (9)	12.9 (11)	
Skin	0.9 (2)	2.9 (2)	
*Candida* species:			
*C. albicans*	25.8 (70)	74.8 (63)	
*C. glabrata*	2.6 (7)	14.8 (12)	0.93
*C. parapsilosis*	2.3 (6)	10.7 (9)	
Other species	6.0 (16)	19.2 (16)	
Polyfungal infection	2.3 (7)	5.7 (5)	1.00
R to fluconazole or NS to echinocandins	5.7 (15)	211 (18)	0.76
Prior antifungal exposure	4.0 (11)	9.1 (8)	0.51
IDC	31.8 (87)	86.6 (73)	0.08

Abbreviations: BSI, bloodstream infection; COPD, chronic obstruction pulmonary disease; HIV, human immunodeficiency virus; HSCT, hematopoietic stem cell transplantation; ICU, intensive care unit; IPWT, inverse probability weighting treatment; IQR, interquartile range; LOS, length of stay; NS, nonsusceptible; SD, Standard Deviation; SOT solid organ transplant. Data are presented as absolute numbers (%) unless otherwise indicated. Variables used in the estimation of the propensity score. ** weighted chi^2^ or weighted Fisher’s exact test and weighted Student’s *t* or weighted Mann-Whitney U test, number may not be integer because of weighting.

**Table 4:** Unadjusted (a) and stabilized IPWT (b) analysis of outcome associated with ESCMID adherence.

(a)				
	Unadjusted			
	*n*	Adherent Case	Nonadherent Case	*p* *
Death at day 30, *n* (%)	165	6 (14)	40 (33)	0.014
LOS (days), median (IQR)	159	36.0 (23.0–56.0)	33.5 (18.0–58.5)	0.41
**(b)**				
	**Stabilized IPWT**			
	**wn**	**Adherent Case**	**Nonadherent Case**	*p* **
Death at day 30, *n* (%)	156.1	3.4 (9.4)	42.4 (35.5)	0.004
LOS (days), median (IQR)	149.4	38.0 (23.0–56.0)	30.0 (15.0–53.0)	0.09

Abbreviations: IQR, interquartile range; LOS, length of stay. Data are presented as absolute numbers (%) unless otherwise indicated. * chi^2^ or Fisher’s exact test for qualitative variables and Student’s *t* or Mann-Whitney U test for quantitative variables. ** weighted chi^2^ or weighted Fisher’s exact test and weighted Student’s *t* or weighted Mann-Whitney U test, number may not be integer because of weighting.

**Figure A1:**
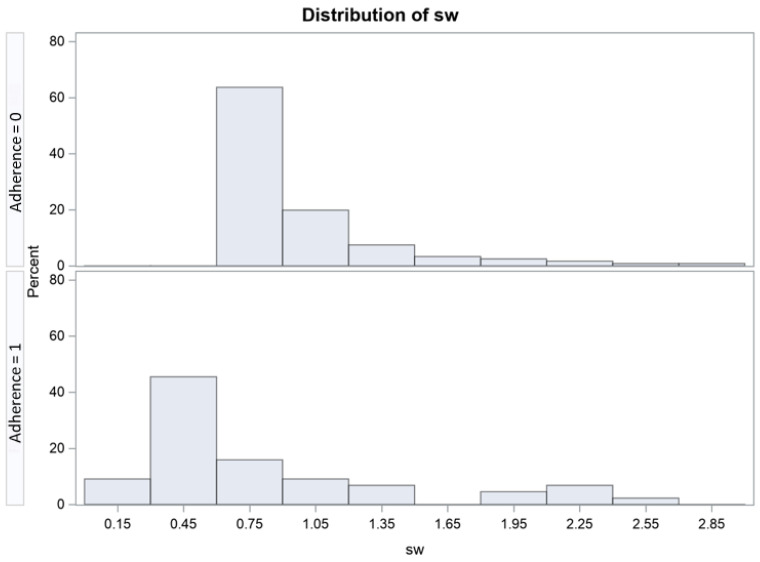
Distribution of weighting in pseudo-population according to ESCMID recommendations adherence.

## Data Availability

Data available on request due to restrictions privacy.
